# Determination of 2′-Fucosyllactose and Lacto-N-neotetraose in Infant Formula

**DOI:** 10.3390/molecules23102650

**Published:** 2018-10-16

**Authors:** Sean Austin, Denis Cuany, Julien Michaud, Bernd Diehl, Begoña Casado

**Affiliations:** 1 Nestlé Research, Vers-Chez-Les-Blanc, 1000 Lausanne, Switzerland; denis.cuany@rdls.nestle.com (D.C.); julien.x.michaud@gsk.com (J.M.); begona.casado@nestle.com (B.C.); 2 Current address: GSK Consumer Healthcare S.A., Route de l’Etraz 2, 1260 Nyon, Switzerland; 3 Spectral Services, Emil-Hoffmann Strasse 33, D-50996 Köln, Germany; diehl@spectralservice.de; 4 Société des Produits Nestlé SA, Nutrition SBU, Rue d’Entre-deux-Villes 10, 1814 La-Tour-De-Peilz, Switzerland

**Keywords:** human milk oligosaccharides, liquid chromatography, infant formula, 2′-FL, LNnT

## Abstract

Human milk oligosaccharides (HMO) are the third most abundant solid component of human milk. It is likely that they are responsible for at least some of the benefits experienced by breast-fed infants. Until recently HMO were absent from infant formula, but 2′-fucosyllactose (2′-FL) and lacto-N-neoteraose (LNnT) have recently become available as ingredients. The development of formula containing these HMO and the quality control of such formula require suitable methods for the accurate determination of the HMO. We developed two different approaches for analysis of 2′-FL and LNnT in formula; high performance anion exchange chromatography with pulsed amperometric detection (HPAEC-PAD) and hydrophilic interaction liquid chromatography with fluorescence detection (HILIC-FLD). In lab trials using blank formula spiked with the two oligosaccharides, both approaches worked well with recoveries of 94–111% (HPAEC-PAD) and 94–104% (HILIC-FLD) and RSD (iR) of 2.1–7.9% (HPAEC-PAD) and 2.0–7.4% (HILIC-FLD). However, when applied to products produced in a pilot plant, the HPAEC-PAD approach sometimes delivered results below those expected from the addition rate of the ingredients. We hypothesize that the oligosaccharides interact with the formula matrix during the production process and, during sample preparation for HPAEC-PAD those interactions have not been broken. The conditions required for labeling the HMO for detection by the FLD apparently disrupt those interactions, and result in improved recoveries. It is likely that both analytical approaches are appropriate if a suitable extraction process is used to recover the HMO.

## 1. Introduction

Human milk oligosaccharides (HMO) are the third most abundant solid component of human milk after lactose and fats. HMO are nondigestible oligosaccharides and, among other things, are believed to be implicated in protecting the infant from infections, and in the establishment of a healthy gut microbiota [1,2,3,4,5]. Although more than 150 different HMO have been identified [6], quantitative data only exists for about 30 of the HMO in milk [7].

The HMO can be roughly classified in to three different types; fucosylated HMO, sialylated HMO, and the core HMO. This is not a rigid separation of structures, since certain HMO can be both fucosylated and sialylated and thus can be classified in to two of the groups. Within the fucosylated HMO, 2′-fucsyllactose (2′-FL) is typically the most abundant, and has been measured at levels up to 8 g/L [8], but most typically is present in milk at levels between 2 and 3 g/L [7]. The potential health benefits of fucosylated HMO have been studied and there is good evidence suggesting that they provide protection against diarrhea [9,10,11]. Lacto-N-neotetraose (LNnT) is one of the more abundant core structures, which can be found without further modification in human milk. It has been detected at levels between 0.010 and 4.1 g/L [12,13], and is typically found at levels between 0.3 and 1.1 g/L [7]. Levels of both LNnT and 2′-FL are quite dynamic, with large variations in concentrations observed between individuals, and depending on stage of lactation.

Despite their abundance in human milk, HMO have not historically been a component of infant formula, largely because their function was unclear and because they were difficult to obtain; they are not present in significant quantities in the milk of farmed animals and routes to produce them on an industrial scale have been prohibitively expensive. However, as science has progressed the potential roles of HMO have started to be elucidated [1,2]. Concomitantly, routes to synthesize HMO on a large scale and at reasonable cost have been developed [14]. The supply of HMO is now such that they can be introduced to infant formula [15,16,17], and 2016 saw the first launches of formula containing 2′-FL in the USA and 2′-FL and LNnT in Europe.

During the development of formula containing HMO, methods had to be developed to determine HMO concentration in the finished product. Apart from being used for quality control purposes to ensure labeling compliance, the methods were also needed to confirm the levels of HMO in products destined for tolerance and safety trials, efficacy trials, and to ensure HMO stability during the formula production process. In this paper we describe two chromatographic methods for the determination of 2′-FL and LNnT in infant formula. One method is based on high performance anion exchange chromatography with pulsed amperometric detection (HPAEC-PAD) and the other on hydrophilic interaction chromatography with fluorescence detection (HILIC-FLD). Both techniques have previously been applied for the analysis of HMO in human milk [18,19]. Applied to spiked infant formula, both methods performed well. However, applied to some formula produced on pilot or industrial plants the HPAEC-PAD method returned lower results, while the HILIC-FLD method returned results more in line with expectations. We suspect the sample preparation procedure for the HILIC-FLD method disrupts interactions between HMO and the product matrix, those interactions are not disrupted using the more simple “dilute and shoot” approach used with HPAEC-PAD.

## 2. Results

In order to determine the quantity of oligosaccharides added to the formula by chromatography it was necessary to obtain good quality, quantitative, analytical standards. At the start of this study such standards were not readily available. The HMO ingredients themselves were therefore used as the standards. In order to establish the purity and moisture content of the ingredients, quantitative nuclear magnetic resonance spectroscopy (qNMR) was employed. Using the well-characterized material it was possible to develop chromatographic methods for the analysis and validate them.

### 2.1. Quantitative Nuclear Magnetic Resonance Spectroscopy (qNMR)

Several batches of oligosaccharide ingredients were analyzed by qNMR to establish the content of oligosaccharide in the ingredient powder (Table 1). The analysis of multiplicity and chemical shift of the ^1^H-NMR and the ^13^C-NMR signals were in accordance with the structures of LNnT and 2′-FL and checked by 2-dimensinal heteronuclear single quantum coherence (HSQC) and heteronuclear multiple bond correlation (HMBC) experiments. In most cases the ingredient powders contained more than 90% of the oligosaccharide, and the remaining mass of powder was predominantly moisture (Table 1). The data obtained from qNMR enabled us to use the ingredients as quantitative standards during validation studies. Aliquots of some batches were used as analytical standards, while the remaining material was used as material for laboratory spiking experiments and for pilot plant trials.

### 2.2. Determination of 2′-FL and LNnT by HPAEC-PAD

**Figure 1:**
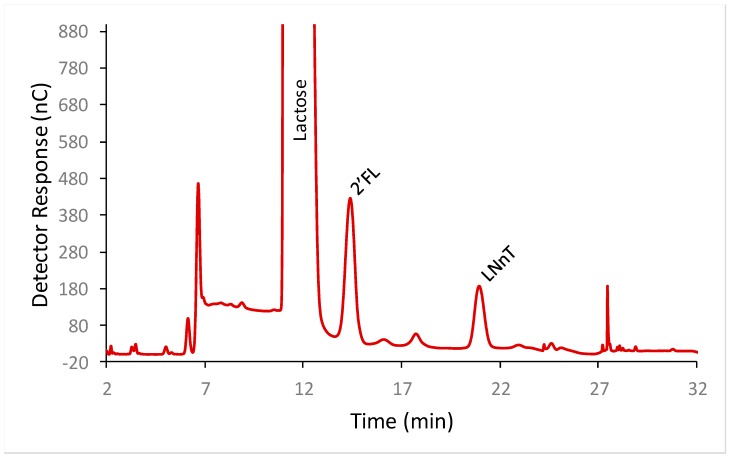
Separation of 2′-fucosyllactose (2′-FL) and Lacto-N-neotetraose (LNnT) from each other and from lactose in infant formula by high performance anion exchange chromatography with pulsed amperometric detection (HPAEC-PAD).

2′-FL and LNnT were well separated from each other and from lactose by HPAEC-PAD (Figure 1). To determine the calibration model, solutions of LNnT and 2′-FL were prepared at nine different concentrations (from 29 to 118 mg/L for LNnT and from 60 to 222 mg/L for 2′-FL) in triplicate, and all the solutions were injected on the HPAEC-PAD system. The detector response was found to be linear for both oligosaccharides. For LNnT the slope of the curve was 0.97 and the Y intercept was at 0.688, with an r^2^ of 0.999. For 2′-FL the slope of the curve was 0.46 and the Y-intercept was −1.91 with an r^2^ of 0.998.

The limit of detection (LoD) and limit of quantification (LoQ) were determined by analyzing oligosaccharide standards at five different concentrations in triplicate between 1 and 20 µg/mL. Plots of signal to noise (S/N) ratio against concentration were prepared for 2′-FL and LNnT. LoDs at S/N ratio of 3 were determined to be 4.33 µg/mL for 2′-FL and 0.48 µg/mL for LNnT. LoQs at S/N ratio of 10 were determined to be 12.8 µg/mL for 2′-FL and 1.51 µg/mL for LNnT. Starting with a sample amount of 2 g dissolved in 100 mL, the LoD and LoQ of 2′-FL in a product were estimated to be 0.02 g/100 g and 0.06 g/100g respectively, and the LoD and LoQ of LNnT in a product were estimated to be 0.003 g/100 g and 0.008 g/100g respectively.

The trueness and precision of the HPAEC-PAD method were assessed by analyzing two different infant formula samples spiked with the oligosaccharides at three different levels (Table 2). Spike-recoveries were in the range 94 to 99% for 2′-FL and 98 to 111% for LNnT. Repeatabilities were in the range of 0.47 to 5.2% for 2′-FL, and 1.1 to 5.6% for LNnT and intermediate reproducibilities were in the range 2.2 to 5.7% for 2′-FL and 2.1 to 7.9% for LNnT.

### 2.3. Determination of 2′-FL and LNnT by HILIC-FLD

**Figure 2:**
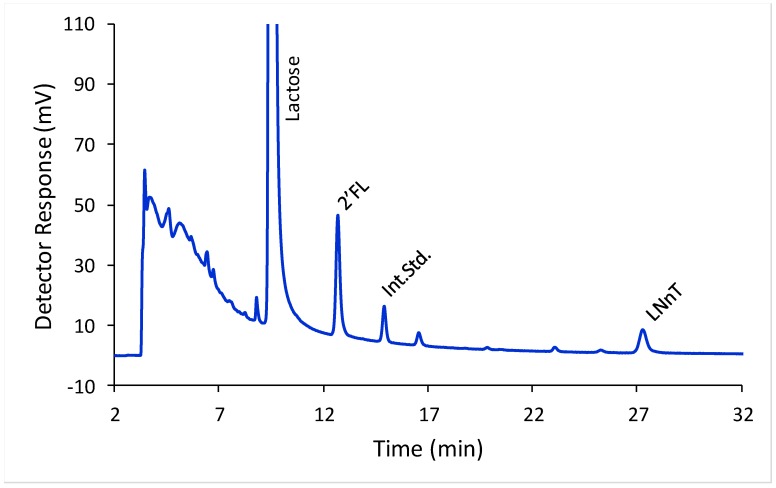
Separation of 2′-fucosyllactose (2′-FL) and Lacto-N-neotetraose (LNnT) from each other and from lactose in infant formula by HILIC-FLD, using laminaritriose as an internal standard (Int. Std.).

2′-FL and LNnT were well separated from each other, from lactose, and from the laminaritriose internal standard using HILIC-FLD (Figure 2). To investigate the calibration model we split the concentration range for both HMO in to two ranges, a low range (covering 2′-FL concentrations from 5 to 293 mg/L and LNnT concentrations from 7 to 426 mg/L) and a high range (covering 2′-FL concentrations from 244 to 2931 mg/L and LNnT concentrations from 355 to 4255 mg/L). For the low range, oligosaccharide solutions were prepared at nine different concentrations in triplicate. For the high range, oligosaccharide solutions were prepared at seven different concentrations in triplicate. In all cases a linear model described the relationship between detector response and concentration. For 2′-FL at the low range the slope was 0.008 and Y-intercept −0.001, with an r^2^ of 0.998. For 2′-FL at the high range the slope was 0.008 and Y-intercept −0.150, with an r^2^ of 0.994. For LNnT at the low range the slope was 0.005 and Y-intercept −0.016, with an r^2^ of 0.997. For LNnT at the high range the slope was 0.005 and Y-intercept −0.188, with an r^2^ of 0.994.

LoD and LoQ were determined by analyzing four different formula powder products and one ready-to-feed liquid product without the addition of HMO, and determining the chromatographic baseline noise at the retention time of 2′-FL and LNnT. Amounts of HMO required to generate a signal 10 times higher than the baseline noise were assigned as LoQ and amounts of HMO required to generate a signal 3 times higher than the baseline noise were assigned as LoD (for powder products the product with the highest values was assigned as the general LoD or LoQ). For 2′-FL in powdered products a small interfering peak was present close to the expected retention time of 2′-FL. In this case the peak area of the interfering peak was used as the baseline noise, and concentrations of 2′-FL giving rise to a peak area of 10 times or 3 times that of the interference peak were assigned as LOQ and LoD, respectively.

For powdered products, the LoDs of 2′-FL and LNnT were estimated to be 0.11 g/100 g and 0.07 g/100 g, respectively, and for liquid products the LoD for 2′-FL and LNnT were estimated to be 0.01 and 0.03 g/L, respectively. For powdered products, the LoQs of 2′-FL and LNnT were estimated to be 0.37 g/100 g and 0.13 g/100 g, respectively, and for liquid products, the LoQs for 2′-FL and LNnT were estimated to be 0.04 g/L and 0.11 g/L, respectively.

Trueness and precision of the method were assessed by analyzing four different infant formula powders spiked with different levels of 2′-FL and LNnT and by analyzing a ready to feed (RTF) formula spiked at three different levels with 2′-FL and LNnT (Table 2). Spike-recoveries were in the range 94 to 101% for 2′-FL and 94 to 104% for LNnT. Repeatabilities were in the range of 1.2 to 2.2% for 2′-FL, and 1.2 to 2.6% for LNnT and intermediate reproducibilities were in the range 2.0 to 7.4% for 2′-FL and 2.1 to 4.9% for LNnT, which are similar to the performance of the HPAEC-PAD method.

### 2.4. Determination of 2′-FL and LNnT in Pilot Plant and Commercial Samples

Ten different formulae produced on pilot or industrial plants were analyzed using both approaches (Table 3). Surprisingly, for eight of the samples the HPAEC-PAD method returned results which were lower than those from the HILIC-FLD methods, and were also lower than the target concentrations where known. However for two of the samples (Formulas (5) and (6) in Table 3), the HPAEC-PAD method returned results similar to or higher than those from the HILIC-FLD.

## 3. Discussion

When we started developing methods for the analysis of HMO, we used oligosaccharide standards purchased off the shelf from laboratory chemical suppliers. However, we rapidly ran into problems achieving oligosaccharide recoveries well in excess of 100%. Troubleshooting exercises suggested that the purity of the standards was significantly less than the purity of the bulk material produced as ingredients. We therefore decided to use the 2′-FL and LNnT ingredients as our standards, but required a way to confirm the purity of the material. This was achieved using qNMR. Good quality HMO standards with qNMR data are not readily available from laboratory chemical vendors, but they can be obtained from some suppliers if specifically requested. Acquiring such standards remains something of a challenge. However, as more HMO become available as ingredients it is likely that good quality quantitative standards will become more readily obtainable.

We initially developed the method for 2′-FL and LNnT analysis on HPAEC-PAD, since it is a well-established technique for the analysis of oligosaccharides, and sample preparation is normally very simple. Validation of the HPAEC-PAD method was performed by dry mixing the HMO in to commercially available formula, and analyzing on six different days in duplicate. Method trueness and precision were both acceptable (Table 2). When we received samples from a pilot plant for analysis the results obtained by HPAEC-PAD were lower than expected from the addition rate (Table 3; formulas (1)–(4)). Investigations at the pilot plant confirmed that the correct addition rates had been applied, and it was not expected that there would be significant degradation of HMO under the processing conditions used. As part of the investigations in the analytical lab, we tried to analyze the samples using an alternative method. Since we were running galactooligosaccharide analysis at the time using HILIC-FLD [20], we decided to apply that method to HMO. We used the same internal standard (laminaritriose), sample preparation protocol, and LC conditions for the HMO analysis as we used for GOS analysis. Unfortunately, under those conditions, the 2′-FL co-eluted with the internal standard, thus it was not possible to quantify 2′-FL, and the LNnT had to be quantified using an external calibration curve. Nevertheless, the measured LNnT concentrations were close to the expected content of 0.40 g/100 g (Table 3. Formulas (1)–(4)). This led us to abandon the HPAEC-PAD method, and start development on the HILIC-FLD method.

The HILIC-FLD method was adapted from the GOS method by changing from HLPC to UHPLC, and adapting the column temperature and gradient conditions to avoid co-elution of 2′-FL with the internal standard. The HILIC-FLD method was also validated by dry-mixing HMO in to existing formula products. The validation data indicated that the HILIC-FLD method performed at least as well as the HPAEC-PAD method on spiked samples (Table 2). Two pilot plant samples were also included in the validation to assess precision (Table 2) in samples which were expected to be more homogenous than the dry-mixed lab samples. The RSD(r) and RSD(iR) determined on the pilot plant samples were similar to the samples from spiking experiments at 5% or less.

A selection of commercial and pilot plant samples was analyzed by both the HILIC-FLD method and the HPAEC-PAD method (Table 3). In general, the results obtained by HILIC-FLD tended to be higher than those obtained by HPAEC-PAD. We hypothesize that there are some interactions between the analytes and the matrix which may be initiated during the industrial production process. That interaction is not completely disrupted when samples are simply extracted in warm water, and thus the analysis by HPAEC-PAD seems to underestimate 2′-FL and LNnT. The more aggressive conditions used during the oligosaccharide labeling procedure (using low pH and DMSO as solvent) seem to disrupt those interactions, and thus the results from the HILIC-FLD method are more in line with expectations. However, as can be seen from data on commercial and pilot plant samples (Table 3), the HPAEC-PAD method does not always underestimate the content of the HMO (see formula (5) and (6) in Table 3). Thus the hypothesis is not universal for all products, and is probably dependent on the product recipe and specific processing conditions.

## 4. Materials and Methods 

### 4.1. Samples

Infant formula samples were obtained from Nestlé Product Technology Centre (Konolfingen, Switzerland), from Wyeth Nutrition (Askeaton, Ireland), or from Nestlé Nutrition (Vevey, Switzerland).

### 4.2. Chemicals

All water was deionized (18 MΩ) produced by a Milli-Q system (Merck Millipore, Darmstadt, Germany), sodium hydroxide solution (50%) was from JT Baker (Deventer, The Netherlands), 2′-FL and LNnT were obtained from Glycom (Hørsholm, Denmark), all other chemicals were of analytical grade or higher and sourced from Sigma-Aldrich/Merck, Darmstadt, Germany.

### 4.3. Determination of 2′-FL and LNnT Purity by Quantitative NMR

NMR measurements were performed on Bruker Avance III 600 spectrometer (Bruker, Karlsruhe, Germany) equipped with a QNP cryo probe head and a Bruker automatic sample changer (B-ACS 120) or on a Bruker Avance III HD 500 spectrometer equipped with a BBO prodigy cryo probe and a Bruker automatic sample changer (B-ACS 120).

For quantification of oligosaccharides, appropriate amounts of the oligosaccharide and of nicotinic acid amide (NSA) internal standard were weighed exactly, dissolved in 1 ml D2O, and measured. Integrated signals of the oligosaccharide and of the internal standard were used for calculation. The ratio of integrals per atom corresponds to the molar ratio of the compared substances.

The amount of water in the samples was determined by ^1^H-NMR in DMSO-*d*_6_. The water signal appears as a separated signal in DMSO and no chemical exchange was observed with the OH protons of the oligosaccharides. The ratio of the water signal representing 2 protons per molecule and a suitable signal of the oligosaccharide (e.g., a hydroxyl resonance with 1 proton per molecule) were used to calculate the molar ratio, after correcting for residual water present in the DMSO solvent. Using the different molecular weights of water and the corresponding oligosaccharide it was possible to calculate the mass balance.

### 4.4. Determination of 2′-FL & LNnT in Formula by HPAEC-PAD

The infant formula sample (2 g) was mixed with water (70 mL) and heated at 70 °C for 25 min with constant stirring. The sample was then clarified by the addition of Carrez I solution (1 mL) followed by the addition of Carrez II solution (1 mL) and sodium hydroxide (1 mol/L; 0.7 mL) and made up to 100 mL with water in a volumetric flask. A portion of the clarified solution was filtered through a 0.22 µm nylon membrane filter in to a LC autosampler vial.

The oligosaccharides were separated on a CarobPac PA1 column (4 × 250 mm) using an ICS 3000 high performance anion exchange chromatography system composed of an autosampler, quaternary gradient pump, column oven and pulsed electrochemical detector with a gold working electrode and silver/silver chloride reference electrode (all from Thermo Fisher Scientific, Waltham, MA, USA). Separation was achieved using the gradient described in Table 4 at a flow of 1 mL/min and column temperature of 30 °C. Injection volume was 10 µL.

### 4.5. Determination of 2′-FL and LNnT in Formula by HILIC-FLD

The infant formula sample (1 g for powder products, 14 mL for liquid products) was mixed with 70 mL of water and heated at 70 °C for 25 min with constant stirring. The cooled solution was diluted to 100 mL with deionized water in a volumetric flask. An aliquot (500 µL) of this solution was transferred to a plastic microtube and laminaritriose solution (0.3 µmol/mL, 200 µL) was added. An aliquot (20 µL) of this solution was transferred to another microtube, and labeling reagent (2-aminobenzamide (0.35 mol/L) + sodium cyanoborohydride (1.0 mol/L) in DMSO containing 30% acetic acid; 200 µL) was added. After mixing the sealed tube was placed in a water bath at 65 °C for 2 h. After cooling, the mixture was diluted with acetonitrile/water (7/3; 1500 µL), particles were removed by centrifugation (10,000× *g*; 5 min), and a portion of the liquid was transferred to a liquid chromatography (LC) autosampler vial. The labeled oligosaccharides were separated by HILIC on a BEH Glycan column (1.7 µm, 2.1 × 150 mm) preceded by a BEH amide guard column (1.7 µm, 2.1 × 50 mm) both from Waters Corporation (Milford, CT, USA). A 2-way 10-port valve was connected between the guard and analytical columns, this was used to direct the eluent from the guard column to waste while excess labeling reagents were eluted, the valve was then switched to direct the flow through the analytical column for separation of the oligosaccharides. The analytical column was held at 60 °C, and the guard column was kept at room temperature (20–25 °C). Separation was achieved using a gradient of ammonium formate (100 mol/L; pH 4.4) and acetonitrile (Table 5). The eluted oligosaccharides were detected by fluorescence using an excitation wavelength of 330 nm and an emission wavelength of 420 nm. The LC system was an Ultimate 3000 RS, with an upper pressure limit of 1000 Bar, connected to a RF2000 fluorimeter (all from Thermo Fisher Scientific), the injection volume was 1 µL.

### 4.6. Method Validation

To check the calibration model of both methods, three independent calibration curves were prepared, each containing at least nine different HMO concentrations. For both analytes, a linear calibration model was applied. The fit of the calibration model was checked by determination of r^2^ and using plots of the residuals.

For the HPAEC-PAD method, limits of detection (LoD) and quantification (LoQ) were determined by analyzing solutions of the HMO at low concentrations, and measuring the signal to noise (S/N) ratio. Concentrations resulting in a S/N ratio of 3 were designated as LoD and those resulting in a S/N ratio of 10 were designated as LoQ. From those concentrations and the procedure for sample preparation is was possible to back-calculate the equivalent LoD and LoQ in a formula powder.

For the HILIC-FLD method, a similar approach as for HPAEC-PAD was followed, however multiple analyses of blank formula were used to estimate the noise level for the chromatogram. Concentrations of HMO resulting in a S/N ratio of 3 were designated as LoD and those resulting in a S/N ratio of 10 were designated as provisional LoQ. These LoDs and LoQs were then compared to the lowest point on the calibration curve, and the highest value (from all the S/N experiments or from the lowest point on the standard curve) was assigned as the definitive LoD or LoQ.

To check the accuracy and precision of the HPAEC-PAD method, two infant formula samples (a whey-predominant formula with intact protein, and a whey-predominant formula with partially hydrolyzed proteins) which did not contain any nondigestible oligosaccharides were spiked (dry blended) with LNnT and 2′-FL, each at three different concentrations and the samples analyzed in duplicate on six different days.

To check the accuracy and precision of the HILIC-FLD method, one infant formula powder (a whey-predominant formula with partially hydrolyzed protein) which did not contain any nondigestible oligosaccharides was spiked with LNnT and 2′-FL at one level. One infant formula powder (a whey-predominant formula with intact protein) which did not contain any nondigestible oligosaccharides was spiked with LNnT and 2′-FL at three different levels. One infant formula powder, and one follow-up formula powder (both whey-predominant formula with intact protein) containing fructooligosaccharides (FOS) were spiked with LNnT and 2′-FL at three different levels. One ready-to-feed formula (whey-predominant formula with intact protein) containing FOS was spiked with LNnT and 2′-FL at three different levels. Spikes in powder products were performed by dry blending in the lab. All samples were analyzed in duplicate on six different days.

Method accuracy was determined by comparing the HMO levels measured with the amount of HMO that had been spiked in the formula. Method precision was determined by estimating the relative standard deviation of the results determined under repeatability (RSD(r)) or intermediate reproducibility (RSD(iR)) conditions.

All data were processed using the in-house statistical program QStat.net using the equations that have been previously described [13].

## 5. Conclusions

2′-FL and LNnT can be accurately determined in infant formula using HILIC-FLD after labeling the oligosaccharides with 2AB. The labeling reaction probably disrupts interactions between the analytes and the matrix, which may be responsible for the lower results sometimes observed when HPAEC-PAD is used for the analysis of samples coming from pilot or industrial plants.

## Figures and Tables

**Table 1:** Human milk oligosaccharides (HMO) content of different batches of HMO.

HMO	Batch	HMO Content of Ingredient (%)	Moisture Content of Ingredient (%)
LNnT	A	91.9	n.d.
LNnT	B	90.6	9.0
LNnT	C	94.5	5.1
LNnT	D	95.6	4.8
LNnT	E	95.5	4.7
LNnT	F	95.5	4.8
LNnT	G	90.8	9.1
2′-FL	A	89.3	n.d.
2′-FL	B	99.3	n.d.
2′-FL	C	99.1	0.5
2′-FL	D	97.0	2.6
2′-FL	E	96.1	3.2
2′-FL	F	96.0	2.9
2′-FL	G	95.7	2.4
2′-FL	H	89.7	3.5

2′-FL = 2′-fucosyllactose; LNnT = Lacto-N-neotetraose; n.d. = not determined.

**Table 2:** Performance of the method determined during validation.

Method	Matrix	Spike (g/100 g)		Measured (g/100 g)		Recovery (%)		RSD(r) (%)		RSD (iR) (%)	
		2′-FL	LNnT	2′-FL	LNnT	2′-FL	LNnT	2′-FL	LNnT	2′-FL	LNnT
HPAEC-PAD	IF powder with intact protein	0.590	0.204	0.583	0.226	98.9	111	2.7	3.5	4.2	7.2
		0.751	0.357	0.735	0.367	97.8	103	1.7	1.1	2.2	2.1
		0.921	0.510	0.886	0.501	96.2	98.1	0.47	1.2	2.2	3.2
HPAEC-PAD	IF powder with partially hydrolyzed protein	0.590	0.204	0.573	0.222	97.0	109	5.2	5.6	4.3	7.9
		0.751	0.357	0.710	0.363	94.6	102	4.8	4.9	5.7	5.6
		0.921	0.510	0.870	0.499	94.4	97.9	1.6	1.7	2.4	3.7
HILIC-FLD	IF powder with intact protein	0.571	0.194	0.557	0.191	97.5	98.8	1.2	2.1	2.7	2.7
		0.723	0.338	0.702	0.331	97.1	97.9	1.7	1.9	3.3	3.2
		0.880	0.484	0.857	0.473	97.4	97.9	1.9	2.3	2.8	3.1
HILIC-FLD	IF powder with partially hydrolyzed protein	0.822	0.425	0.805	0.397	97.9	93.5	1.7	1.2	2.7	4.4
HILIC-FLD	IF powder with intact protein and FOS	0.573	0.193	0.570	0.188	99.6	97.4	1.8	2.4	2.7	4.7
		0.722	0.384	0.710	0.379	98.3	98.5	1.6	1.5	3.4	2.1
		0.874	0.582	0.855	0.579	97.9	99.5	1.2	1.5	2.6	2.5
HILIC-FLD	FUF powder with intact protein and FOS	0.575	0.191	0.557	0.189	96.8	99.1	2.1	1.8	7.4	4.9
		0.722	0.387	0.695	0.390	96.3	101	2.0	1.6	4.5	2.6
		0.877	0.567	0.824	0.576	93.9	102	2.2	1.4	4.2	2.3
HILIC-FLD	IF RTF with intact protein and FOS	0.541 ^a^	0.222 ^a^	0.548 ^a^	0.224 ^a^	101	101	1.5	2.3	2.6	3.5
		1.06 ^a^	0.546 ^a^	1.06 ^a^	0.568 ^a^	100	104	1.8	2.0	2.0	2.5
		4.17 ^a^	1.05 ^a^	4.19 ^a^	1.09 ^a^	101	104	1.2	2.6	2.2	4.8
HILIC-FLD	IF powder with intact protein and FOS (pilot production)	n/a	n/a	0.785	0.390	n/a	n/a	1.5	2.2	3.3	5.0
HILIC-FLD	FUF powder with intact protein and FOS (pilot production)	n/a	n/a	0.600	0.304	n/a	n/a	1.3	1.8	5.0	3.1

^a^ Values in g/L. Abbreviations: FOS = fructooligosaccharides, FUF = follow-up-formula, IF = Infant formula, RTF = ready to feed.

**Table 3:** Determination of HMO in pilot plant or commercial formulae.

Sample	2′-FL (g/100 g)			LNnT (g/100 g)		
	Target	HILIC	HPAEC	Target	HILIC	HPAEC
Formula (1): Intact Protein	0.800	n/a ^(a)^	0.625	0.400	0.452 ^(a)^	0.329
Formula (2): Intact protein	0.800	n/a ^(a)^	0.611	0.400	0.437 ^(a)^	0.331
Formula (3): Intact protein	0.800	n/a ^(a)^	0.630	0.400	0.379 ^(a)^	0.286
Formula (4): Intact protein	0.800	n/a ^(a)^	0.637	0.400	0.394 ^(a)^	0.288
Formula (5): Intact Protein	0.800	0.822	0.904	0.400	0.402	0.410
Formula (6): Intact Protein	0.800	0.824	0.914	0.400	0.416	0.412
Formula (7): Partially hydrolyzed protein	0.190	0.173	0.141	0	nd ^(b)^	nd ^(b)^
Formula (8): Intact protein	Unk. ^(c)^	0.172	0.147	0	nd ^(b)^	nd ^(b)^
Formula (9): Intact protein	Unk. ^(c)^	0.170	0.123	0	nd ^(b)^	nd ^(b)^
Formula (10): Partially hydrolyzed protein	Unk. ^(c)^	0.708	0.664	Unk.	0.342	0.294

^(a)^ Determined using original GOS method [20] in which 2′-FL co-eluted with the internal standard, thus 2′-FL cannot be determined & LNnT was determined using only an external standard. n/a = not applicable. ^(b)^ nd = not detected ^(c)^ Unk. = Target concentration is unknown.

**Table 4:** Gradient for determination of 2′-FL and LNnT by HPAEC-PAD.

Time (min)	Sodium Hydroxide (mM)	Sodium Acetate (mM)
0	50	0
2.0	50	0
18.0	110	0
18.1	110	12.5
25.0	110	12.5
25.1	150	500
30.0	150	500
30.1	300	0
35.0	300	0
35.1	50	0
40.0	50	0

**Table 5:** Gradient for separation of HMO by hydrophilic interaction liquid chromatography with fluorescence detection (HILIC-FLD).

Time (min)	Flow (mL/min)	%A	%B	Valve
0	0.6	98	2	waste
2.5	0.6	98	2	analyse
3.0	0.6	88	12	analyse
10.0	0.6	88	12	analyse
20	0.6	83	17	analyse
33.0	0.6	83	17	analyse
34.0	0.45	30	70	analyse
37.0	0.45	30	70	analyse
39.0	0.45	90	10	analyse
45.0	0.6	90	10	analyse
45.1	0.6	98	2	waste
46	0.6	98	2	waste

Eluent A = acetonitrile; eluent B = ammonium formate (100 mmol/L, pH 4.4).
